# Under Cover at Pre-Angiosperm Times: A Cloaked Phasmatodean Insect from the Early Cretaceous Jehol Biota

**DOI:** 10.1371/journal.pone.0091290

**Published:** 2014-03-19

**Authors:** Maomin Wang, Olivier Béthoux, Sven Bradler, Frédéric M. B. Jacques, Yingying Cui, Dong Ren

**Affiliations:** 1 Key Laboratory of Insect Evolution and Environmental Changes, Capital Normal University, Beijing, P. R. China; 2 Sorbonne Universités - CR2P - MNHN, CNRS, UPMC-Paris6, Paris, France; 3 Johann-Friedrich-Blumenbach-Institute of Zoology and Anthropology, Georg-August-University Göttingen, Göttingen, Germany; 4 Laboratory of Palaeoecology, Xishuangbanna Tropical Botanical Garden, CAS, Menglun, Mengla, Xishuangbanna, Yunnan, P. R. China; 5 Institute of Geology, Department of Palaeontology, Technical University Bergakademie Freiberg, Freiberg, Germany; USDA-Agricultural Research Service, United States of America

## Abstract

**Background:**

Fossil species that can be conclusively identified as stem-relatives of stick- and leaf-insects (Phasmatodea) are extremely rare, especially for the Mesozoic era. This dearth in the paleontological record makes assessments on the origin and age of the group problematic and impedes investigations of evolutionary key aspects, such as wing development, sexual size dimorphism and plant mimicry.

**Methodology/Principal Findings:**

A new fossil insect species, *Cretophasmomima melanogramma* Wang, Béthoux and Ren sp. nov., is described on the basis of one female and two male specimens recovered from the Yixian Formation (Early Cretaceous, ca. 126±4 mya; Inner Mongolia, NE China; known as ‘Jehol biota’). The occurrence of a female abdominal operculum and of a characteristic ‘shoulder pad’ in the forewing allows for the interpretation of a true stem-Phasmatodea. In contrast to the situation in extant forms, sexual size dimorphism is only weakly female-biased in this species. The peculiar wing coloration, viz. dark longitudinal veins, suggests that the leaf-shaped plant organ from the contemporaneous ‘gymnosperm’ *Membranifolia admirabilis* was used as model for crypsis.

**Conclusions/Significance:**

As early as in the Early Cretaceous, some stem-Phasmatodea achieved effective leaf mimicry, although additional refinements characteristic of recent forms, such as curved fore femora, were still lacking. The diversification of small-sized arboreal insectivore birds and mammals might have triggered the acquisition of such primary defenses.

## Introduction

Plant-insect interactions such as herbivory and crypsis are well documented and understood during the rise of angiosperms, but there is a severe gap of knowledge for pre-angiospermous times, when ‘gymnosperms’ were the dominant form of vegetation [1]. It is assumed that the mid-Mesozoic patterns of plant-insect interactions may be ecologically very similar to the Cenozoic ones, demonstrating an almost complete turnover of major plant and insect lineages. This also applies for stick and leaf insects (Phasmatodea) whose extant forms supposedly underwent a rather recent radiation, in co-evolution with angiosperm plants [2], [3]. Indeed, extant Phasmatodea are characterized by elaborate performance at mimicking angiosperm plant parts, such as leaves, twigs, and bark.

Investigating the origin of this interaction has long been impeded by a lack of positive evidence on the occurrence of actual stem-Phasmatodea during Cenozoic times. The central issue has remained to distinguish them from stem-Orthoptera (i.e. fossil relatives of grasshoppers, crickets, and katydids). This debate is worth being considered with sufficient detail. A landmark in the study of putative Mesozoic stem-Phasmatodea (and stem-Orthoptera) is the contribution by Sharov [4], [5], who (re-)described many fossil taxa he considered closely related to Phasmatodea, including the Xiphopteridea (according to him, including all extant taxa and close fossil relatives), and the extinct Chresmodidea, with roots in the Triassic. It must be noticed here that Sharov also described the Mesozoic family Phasmomimidae, he considered member of the Orthoptera.

Several contributions followed Sharov's opinion on the phasmatodean affinities of the corresponding fossils [6]–[9] (among others; Hennig [10] can be considered ‘hesitant’ to accept Sharov's view). However, according to the survey by Tilgner [11], the body of evidence suggesting phasmatodean affinities of those fossils is mostly based on characters of wing venation which are not exclusive to Phasmatodea, and/or character states which are plesiomorphies with respect to Orthoptera. According to this author, the oldest fossils that can be conclusively assigned to Phasmatodea are very recent (44–49 mya). However, in the same contribution (p. 475) Tilgner admitted that the female operculum, the enlarged abdominal sternum 8, described by Ren [12] in some remarkable Chinese Cretaceous fossils (family Hagiphasmatidae) suggests actual affinities with Phasmatodea.

Meanwhile, a number of fossil taxa were described and assigned to Sharov's Phasmomimidae ([13], [14]; among others). Most of the corresponding taxa were not considered by Tilgner [11]. A broader picture was reached by Gorochov [15] who, relying on Ren's data [12], relocated a large portion of the ‘Phasmomimidea/-oidea’ in the Susumaniidae, which, together with the Hagiphasmatidae, formed the Susumanioidea, within Phasmatodea.

**Figure 1:**
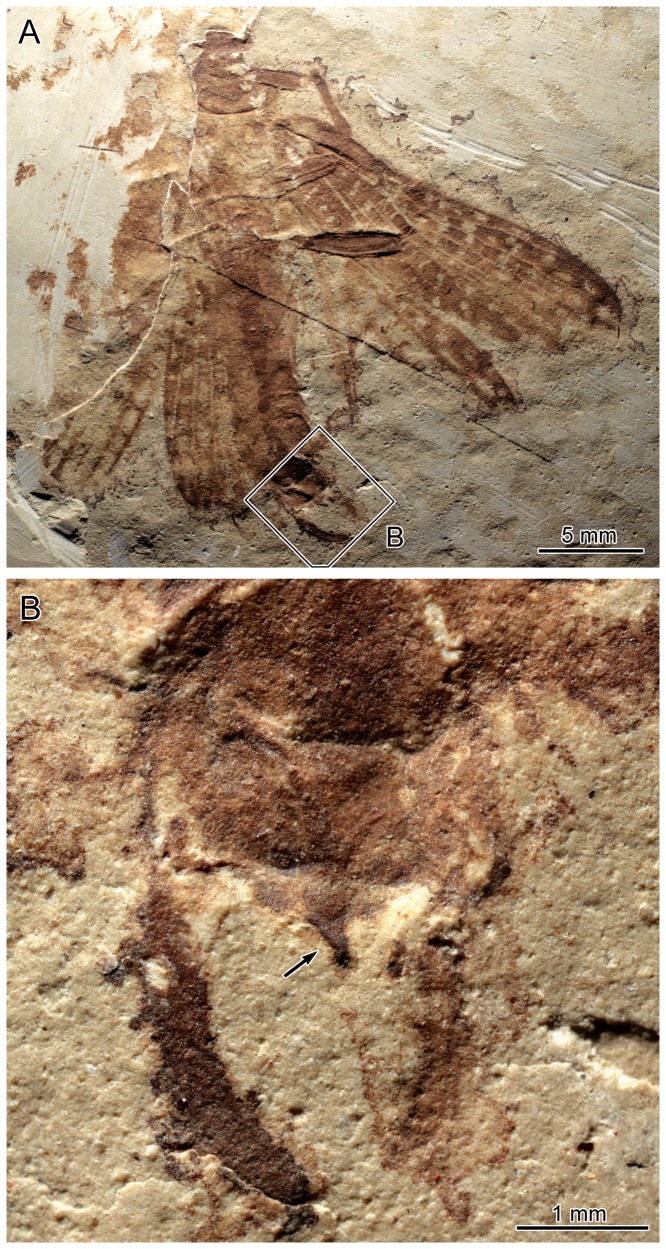
*Renphasma sinica* Nel and Delfosse, 2011. Holotype specimen MNHN A31857, ♂ (negative imprint). A. Habitus. B. Detail of terminalia (as located on A); arrow indicates the vomer terminal hook.

Several more recent accounts can be considered inconclusive. The position of Sharov's Xiphopteridea and Chresmodidea remains uncertain (as for the former, see [15]; as for the latter, see [16]), and so is the situation of a curious Eocene fossil interpreted as a stem-Phasmatodea [17], [18]. However, there have been several important improvements in the identification of Mesozoic stem-Phasmatodea in this century. Nel and Delfosse [19] described a Cretaceous fossil possessing a ‘susumanioid’ wing venation and a vomer (see Figure 1), a clasping structure of the male terminalia, which is a strict apomorphy of Phasmatodea [20]–[22]. Concurrently, Shang et al. [23] described a female specimen possessing the decisive operculum and a male specimen possessing terminalia with ‘elongated elements provided with a thorn pad’ bearing strong similarities with extensions of the 10^th^ tergum common in stick-insects (see [21]). Both specimens are Jurassic in age.

In summary, fossil specimens preserving terminalia have been decisive for the identification of Mesozoic genuine stem-Phasmatodea, but our knowledge of the evolutionary history of the group is yet very limited. In the following we describe a new insect species, recovered from the famous Cretaceous Jehol biota (129±4 mya [24]), known based on male and female specimens. The species can be conclusively considered as a stem-Phasmatodea. The remarkable preservation of the specimens and of the corresponding environment allows the investigation of key aspects of this species' palaeoecology.

## Materials and Methods

### Specimens location

**Figure 2:**
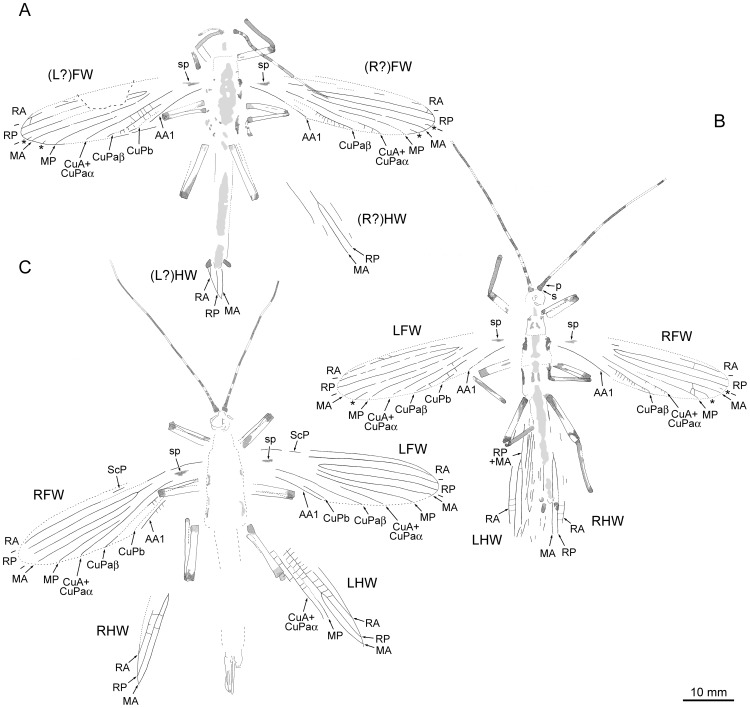
*Cretophasmomima melanogramma* Wang, Béthoux and Ren sp. nov., habitus drawings (* indicate intercalary veins; see text for abbreviations; all at the same scale). A. Specimen CNU-PHA-NN2012003, ♂. B. Holotype specimen CNU-PHA-NN2012002, ♂. C. Specimen CNU-PHA-NN2012001, ♀.

**Figure 3:**
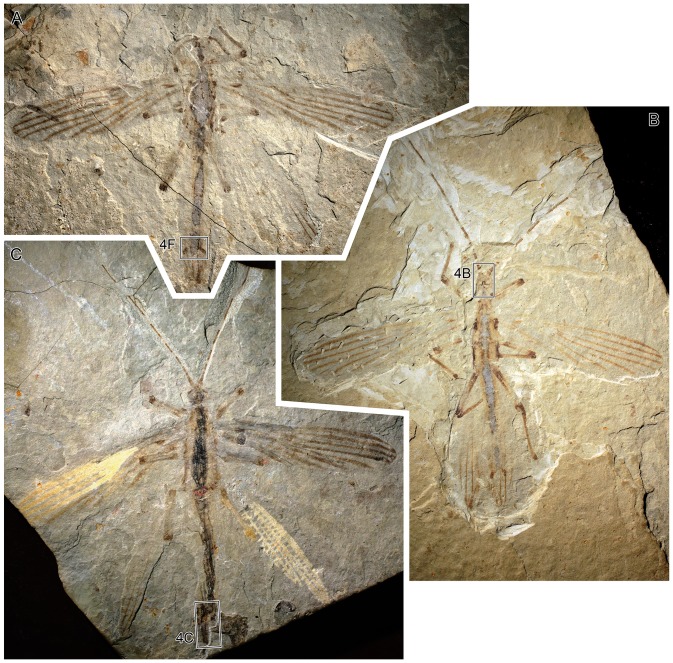
*Cretophasmomima melanogramma* Wang, Béthoux and Ren sp. nov., habitus photographs. A. Specimen CNU-PHA-NN2012003, ♂. B. Holotype specimen CNU-PHA-NN2012002, ♂. C. Specimen CNU-PHA-NN2012001, ♀.

**Figure 4:**
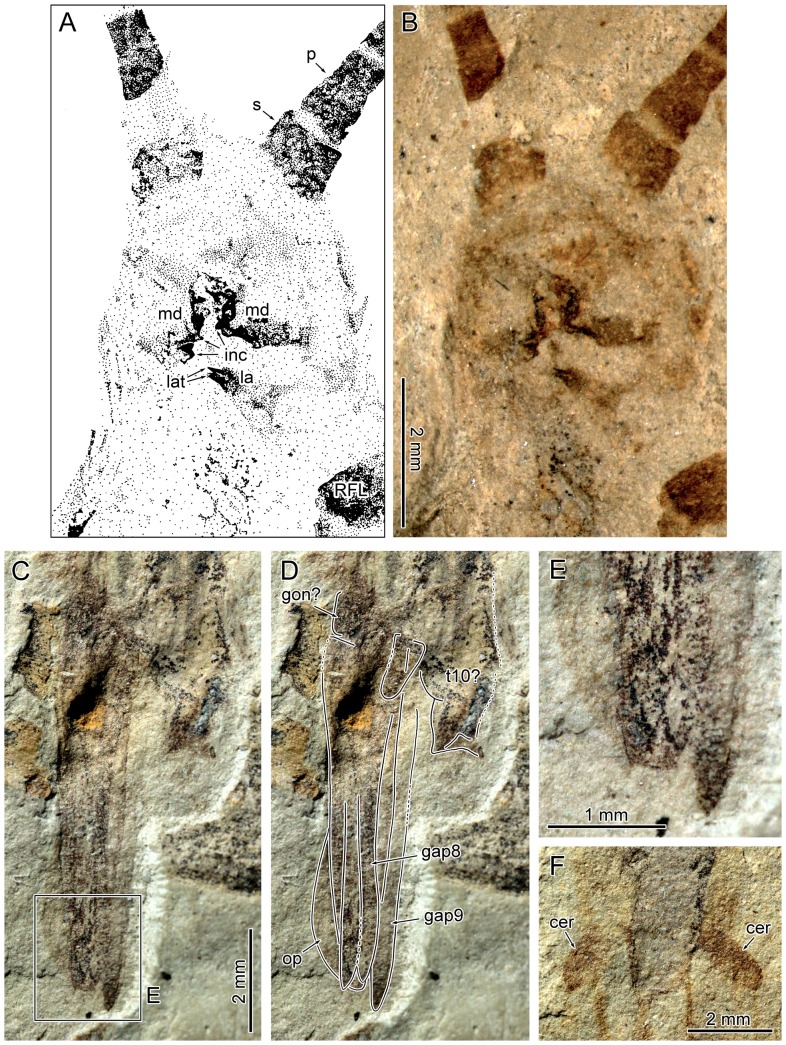
*Cretophasmomima melanogramma* Wang, Béthoux and Ren sp. nov. A–B. Specimen CNU-PHA-NN2012002, ♂, head morphology (see text for abbreviations), as located in Figure 3B. A. Drawing. B photograph. C–F. Terminalia morphology (see text for abbreviations). C–E. Specimen CNU-PHA-NN2012002, ♀, photographs, as located in Figure 3C. C. Photograph with interpretation, to be compared to D and E. F. Specimen CNU-PHA-NN2012002, ♂, as located in Figure 4A.

**Figure 5:**
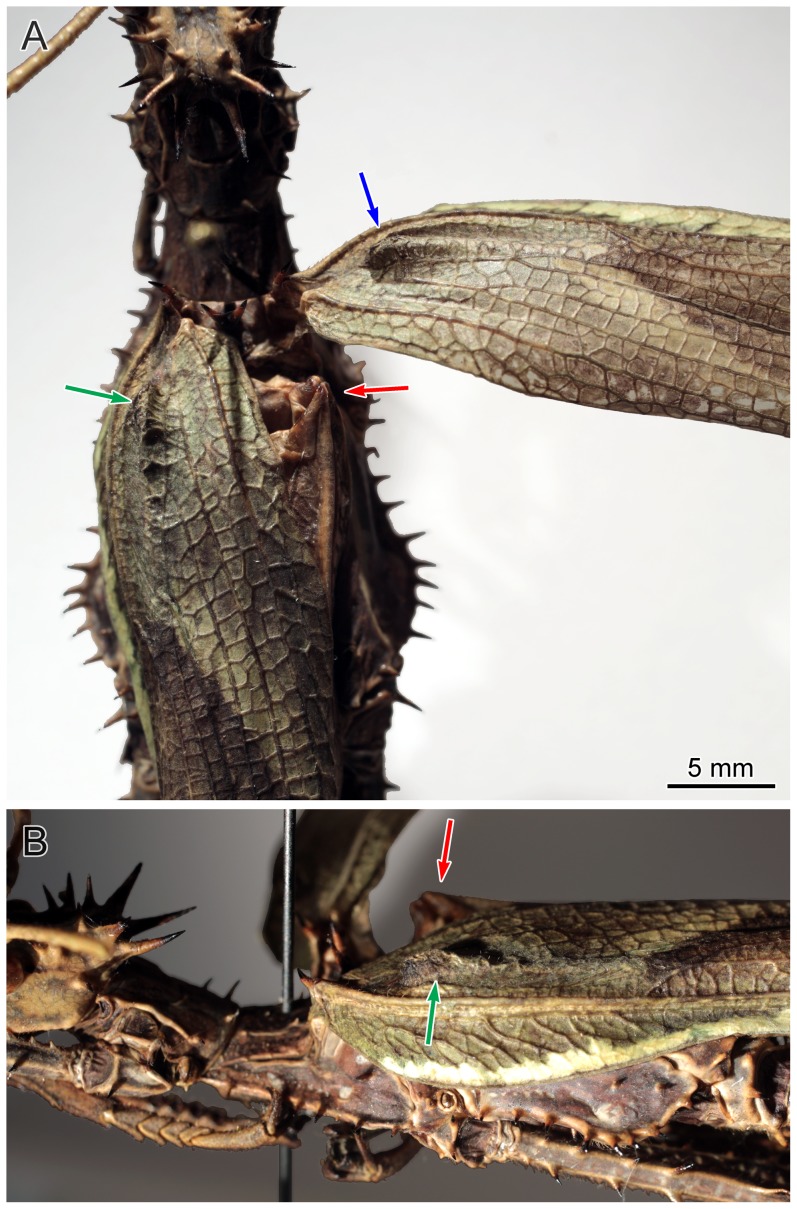
*Heteropteryx dilatata* (Parkinson, 1798), details of the ‘shoulder pad’ (all at the same scale). Shoulder pad of the right forewing and left forewing indicated by a blue and green arrow, respectively. Base of the right hind wing indicated by a red arrow. A. Dorsal view. B. Left lateral view, slightly tilted laterally.

**Figure 6:**
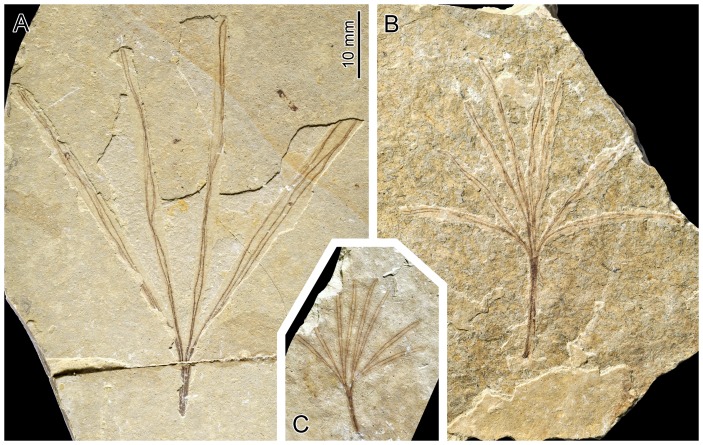
Exemplars of *Membranifolia admirabilis* Sun and Zheng, 2001 in Sun, Zheng, Dilcher, Wang and Mei, 2001 (all at the same scale, and same scale as **Figure 2**). A. Holotype specimen PB19184. B. Specimen PB19196. C. Specimen PB19185.

The three specimens CNU-PHA-NN2012001, CNU-PHA-NN2012002 and CNU-PHA-NN2012003 (represented on Figures 2–4), and described as belonging to a new species, are housed at the Key Lab of Insect Evolution and Environmental Changes, College of Life Sciences, Capital Normal University (acronym CNU-; Beijing, P. R. China). These specimens were collected by the research team of one of the authors (DR), who is also curator of the corresponding collection. The specimen MNHN A31857 (represented on Figure 1), holotype of *Renphasma sinica* Nel and Delfosse, 2011, is housed at the Muséum National d'Histoire Naturelle, Paris (France). One of the authors (OB) obtained a loan of this specimen (curator: A.Nel). The extant specimen represented on Figure 5, belonging to the species *Heteropteryx dilatata* (Parkinson, 1798), belongs to the private collection of one of the authors (OB). It was purchased and is unnumbered. Fossil specimens reproduced on Figure 6, belonging to *Membranifolia admirabilis* Sun and Zheng, 2001 in Sun, Zheng, Dilcher, Wang and Mei, 2001, are housed at the Nanjing Institute of Geology and Palaeontology, Chinese Academy of Sciences (holotype specimen PB19184, and specimens PB19196 and PB19185; Nanjing, P. R. China). One of the authors (FJ) was granted access to these corresponding collections.

### Image acquisition and processing

The specimens CNU-PHA-NN2012001, CNU-PHA-NN2012002 and CNU-PHA-NN2012003 were examined under dry conditions using a Leica DCF 500 dissecting microscope and illustrated with the aid of a drawing tube. Photographs were taken using a Canon EOS 550D digital camera coupled to a Canon 50 mm macro lens (and an extension tube as appropriate), or a Canon MP-E 65 mm macro lens (all lenses equipped with polarizing filters).

### Abbreviations for morphological features

Abbreviations are as follows: ScP, posterior Subcosta; R, Radius; RA, anterior Radius; RP, posterior Radius; M, Media; MA, anterior Media; MP, posterior Media; CuA, anterior Cubitus; CuP, posterior Cubitus; CuPa, anterior branch of CuP; CuPaα, anterior branch of CuPa; CuPaβ, posterior branch of CuPa; CuPb, posterior branch of CuP; AA1, first anterior Analis; md, mandible; la, lacinia; lat, lacinia teeth; inc, incisive; p, pedicel; s, scale; sp, shoulder pad; gon, gonangulum; op, operculum; gap8 and gap9, gonapophysis 8 and 9, respectively; t10, tergum 10; cer, cercus; RFL, right foreleg; LFW, left forewing; LHW, left hind wing; RFW, right forewing; RHW, right hind wing.

### Homology and terminology

As usual in entomology [25], a variety of conjectures of topological homology have been proposed for the wing venation of (stem-)Phasmatodea (e.g. [26]'s vs. [15]'s vs. [19]'s). In agreement between authors, we follow the Zeuner [27] – Sharov [4], [5] – Gorochov [15] conjectures for Orthoptera and Phasmatodea, including modification by Ren [12] (namely, assuming a RP+MA fusion in hind wings of Phasmatodea; followed in [23]), but converted under Béthoux and Nel's scheme for Orthoptera with respect to other winged insects [28], [29]. These last conjectures were challenged by Gorochov [30], but the latter was subsequently addressed [31]. Further comments by Rasnitsyn [32], supporting the alternative conjectures proposed by Rasnitsyn in Gorochov and Rasnitsyn [33], have been discussed elsewhere [34]. The use of an ‘orthopteran’ set of conjectures does not preclude on the supposed affinities of Phasmatodea with Orthoptera. In agreement between the authors, the traditional nomenclatural procedure is followed herein. This does not imply support for this approach on the part of OB (see [35]).

### Nomenclatural acts

The electronic edition of this article conforms to the requirements of the amended International Code of Zoological Nomenclature, and hence the new names contained herein are available under that Code from the electronic edition of this article. This published work and the nomenclatural acts it contains have been registered in ZooBank, the online registration system for the ICZN. The ZooBank LSIDs (Life Science Identifiers) can be resolved and the associated information viewed through any standard web browser by appending the LSID to the prefix “http://zoobank.org/”. The LSID for this publication is: urn:lsid:zoobank.org:pub: A36F858B-FA70-4BE5-84DF-EAAA4F40F9A7. The electronic edition of this work was published in a journal with an ISSN, and has been archived and is available from the following digital repositories: PubMed Central, LOCKSS, Central Library at the Muséum National d'Histoire Naturelle (Paris). An offprint of this work was deposited at the Central Library at the Muséum National d'Histoire Naturelle (Paris, France).

## Systematic Palaeontology

### 

**Order Phasmatodea Jacobson and Bianchi, 1902, superfamily Susumanioidea Gorochov, 1988, family uncertain, genus** ***Cretophasmomima*** **Kuzmina, 1985,** ***Cretophasmomima melanogramma*** **Wang, Béthoux and Ren sp. nov.**

### 

urn:lsid:zoobank.org:pub: A36F858B-FA70-4BE5-84DF-EAAA4F40F9A7

#### General description

Female and male body lengths, from head to tip of terminalia, about 56 mm and 47–51 mm, respectively (male, from head to tip of hind wings, about 50–53 mm); *head:* globular, mandible with incisivi; lacinia with two apical teeth; antenna filiform, at least 37.8 mm and 34.3 mm long in female, and male, respectively, alternating pale and dark areas (areas gradually longer from the base to the apex); *thorax*: prothorax and mesothorax trapeze-shaped, metathorax quadrangular; segment boundaries vaguely discernible, mesothorax and metathorax of equal length, prothorax shorter, legs gressorial, with bases and apices of femora and tibiae dark-colored, all legs of equal length and shape with hind legs only marginally longer the fore and mid legs, fore legs inserted on prothorax directly behind the head, mid and hind legs inserted on meso- and metathorax at each segment's hind margin; tarsus morphology unknown (preserved in none of the specimens, or insufficiently); two pairs of dark areas (herein interpreted as the wing bases) located antero-laterally in the meso- and metathorax; *forewings*: aspect ratio about 4.3; ScP running close to anterior margin; RA simple, straight, with rare anterior veinlets; RP with two branches, forked opposite the first third of wing length; MA and MP simple; basal part of MP weak; intercalary veins in the distal parts of the areas between RP and MA, and between MA and MP; CuA+CuPaα, CuPaβ, CuPb and AA1 sub-parallel, simple; area between CuPb and AA1 very narrow; cross-veins weak and/or not colored, except for those in the distal part, and in the area between CuPaβ and CuPb, which are stronger; CuPaβ and CuPb slightly sigmoidal; CuPb very weak; main veins and intercalary veins surrounded by dark coloration in most of the forewing; Y-shaped dark area (herein interpreted as ‘shoulder pad’) located in the first quarter of wing length; *hind wings*: RP and MA simple, slightly anteriorly bent near apex; location of the point of divergence of RP and MA variable, at least distal with wing mid-length; most of MP weakly sclerotized; in middle part of remigium, cross-veins not reticulated, forming quadrangular cells; hind wings extending beyond terminalia at rest, with the distal-most portion not covered by forewings; in this distal part, main veins surrounded by dark coloration; *abdomen:* distinctly narrower than thorax; *male terminalia:* with two similar dark brown cerci, no visible segmentation; epiproct possibly preserved; *female terminalia:* gonapophysis 8 and 9 elongated, operculum present, slender, as long as gonapophyses.

#### Specimens descriptions

Specimen CNU-PHA-NN2012003 (Figures 2A, 3A, 4F): moderately well-preserved specimen, interpreted as male; probably positive imprint; body length about 46 mm (excluding antennae); *head:* poorly preserved, lobes of both mandibles preserved on inner side, most probably incisivi; antennae incomplete; scape robust, quadrangular, width 0.8 mm, length 0.7 mm; *thorax:* outline and segments delimitation indistinct; legs incompletely preserved; tarsus of the right? mid leg about 1.2 mm long, poorly preserved; *forewings:* elongated, areas along venation brown-colored; intercalary veins between RP and MA, and between MA and MP; CuPb weak (almost invisible in right forewing); Y-shaped dark are (‘shoulder pad’) located in the first quarter of wing length; *left? forewing:* length about 37 mm, width about 10 mm (middle of the wing), area between anterior wing margin and RA with one preserved cross-vein/veinlet in distal part; *right? forewing:* length about 38 mm; width about 8.8 mm (middle of the wing); *hind wings:* very poorly preserved, only a portion of anterior-distal part of right? hind wing interpretable; RP and MA simple, diverging from a common stem; *abdomen:* outline and segments delimitation indistinct; alimentary canal preserved as gray carbonaceous remains; terminalia with two similar dark brown cerci, no visible segmentation.

Specimen CNU-PHA-NN2012002 (holotype; Figures 2B, 3B, 4A, B): well-preserved individual, interpreted as male; positive imprint; body length 46.6 mm (excluding antennae); *head*: globular; inner margins of both mandibles preserved, showing incisivor lobes on both sides (Figure 4A, B); lacinia of right maxilla visible, with two apical teeth; antennae filiform, about 34 mm long as preserved (incomplete), with many segments (at least 14 discernible); scape and pedicel robust, dark brown; scape width 1.0 mm, length 0.8 mm; pedicel trapeze-shaped, width 0.7 mm, length 1.2 mm; *thorax:* prothorax trapeze-shaped, width near the mesothorax 3.8 mm, length 4.1 mm; lengths of mesothorax and metathorax similar, about 5.8 mm; mesothorax trapeze-shaped; metathorax quadrangular, width 5.6 mm; *forewings:* elongated; venation visible mostly along brown-colored areas; ScP not visible; intercalary veins preserved between RP and MA (right forewing), and between MA and MP (both forewings); CuPb very weak (not visible in right forewing); AA1 reaching posterior wing margin opposite the fork of RP; overall, only few cross-veins visible; in distal part, areas surrounding main vein brown-colored; occurrence of a Y-shaped brown area located in the first quarter of wing length, wing anterior half (interpreted as ‘shoulder pad’); *left forewing:* 28.4 mm long as preserved (estimated complete length about 37.1 mm), width 8.7 mm (middle of the wing); area between anterior wing margin and RA with two preserved cross-veins in distal part; *right forewing:* 25.4 mm long as preserved (estimated complete length about 35.8 mm), width 8.8 mm (middle of the wing); *hind wings:* in resting position, partly overlapping, antero-distal part interpretable; preserved length about 24 mm; RP and MA fused for at least 7.1 mm (left hind wing), then separated, near wing mid-length; RP and MA simple, slightly anteriorly bent near apex; *abdomen:* outline and segments delimitation indistinct; alimentary canal preserved as gray carbonaceous remains; terminalia with two similar dark brown cerci, no visible segmentation.

Specimen CNU-PHA-NN2012001 (Figures 2C, 3C, 4C–E): moderately well-preserved individual, interpreted as female; negative imprint; body length 55.6 mm (excluding antennae); *head:* globular, width 3.8 mm; antennae filiform, incomplete; preserved length 36.1 mm/27.6 mm (right/left antenna), with at least 18/16 segments discernible; scape robust, dark brown, width 1.0 mm, length 0.7 mm; pedicel trapeze-shaped, width 0.8 mm (middle of the pedicel), length 0.9 mm; *thorax:* outline indistinct; *forewings:* margins of right forewing and of most of left forewing untraceable; ScP incomplete, only one piece preserved in the first third of wing length; RA slightly bent in distal part, reaching anterior wing margin near apex; Y-shaped brownish area (‘shoulder pad’) located in the first quarter of wing length; *right forewing:* 40.1 mm long as preserved (estimated complete length about 40.9 mm), width 9.8 mm (middle of the wing); left forewing: 35.3 mm long as preserved (estimated complete length about 38.8 mm), width 9.7 mm (middle of the wing); *hind wings:* spread, poorly preserved, only antero-distal part interpretable; preserved length 25.8 mm; RP and MA fused for a least 10.8 mm (left hind wing), then separated, near two third of wing length; RP and MA simple, slightly anteriorly bent near apex; numerous cross-veins; *abdomen:* outline indistinct, alimentary canal preserved as gray carbonaceous remains; terminalia relatively complete, oriented laterally; operculum light-colored, partly hidden by darker gonapophysis 8 and gonapophysis 9; location and shape of cerci and gonangulum obscure.

#### Diagnosis

Main veins and intercalary veins surrounded by dark coloration in most of the forewing, and in distal part of the hind wing.

#### Type material

Specimen CNU-PHA-NN2012002.

#### Locality and horizon

Early Cretaceous (126±4 mya [24]); Yixian Formation; Liutiaogou Village, Ningcheng Country, Inner Mongolia, NE China.

#### Etymology

Specific epithet composed of ‘melano’, Ancient Greek for ‘black’, and ‘gramme’, Ancient Greek for ‘line’; referring to the coloration of forewings, and of hind wing apices.

#### Remarks

The interpretation of specimen CNU-PHA-NN2012001 as a female is straightforward (according to its terminalia; Figure 4C–E). The specimens CNU-PHA-NN2012002 and CNU-PHA-NN2012003 share evident similarities with this specimen, such as body size, and coloration of body parts. Provided that terminalia of these two specimens (Figures 2A–B, 3A–B, 4F) differ from those of the female, it is assumed that they are males, and are conspecific with the CNU-PHA-NN2012001 female.

Assignment of the new species to the superfamily Susumanioidea is straightforward, notably thanks to the proximal origin of RP in forewing, and the proximal location of the first fork of this vein. It is worth noticing here that these diagnostic traits are shared with extant Phasmatodea (compare with forewing venation of *Hereropteryx dilatata* (Parkinson, 1798); see figure 4 in [23]), suggesting that the Susumanioidea is a paraphyletic taxon. If so its members would better be referred to as ‘stem-Phasmatodea’.

Within the ‘Susumanioidea’, the distal location of the divergence point of RP+MA observed in hind wing of the new species, and documented in several members of the Hagiphasmatidae, suggests that the new species belongs to this taxon. However, this character is not documented in many Susumaniidae, making conclusive familial assignment impossible.

The simple CuA+CuPaα in forewings (to ease comparison, it equates Nel and Delfosse [19]'s MP, Ren [12]'s MP (figure 1), MP+CuA_1_ (figure 2), and anterior branch of MP (figure 3), Gorochov [15]'s and Shang et al. [23]'s MP+CuA_1_/CuA1) indicates close relationships with a number of susumanioid genera, e.g. *Cretophasmomima* Kuzmina, 1985, *Promastacoides* Kevan, 1981, and *Cretophasmomimoides* Gorochov, 1988. However, the most recent detailed account on the taxonomy of these genera [14] (and see [15]) proved to lack ground in several cases. For example, the differences between the genera *Cretophasmomima* and *Promastacoides* are considered “not entirely evident” (p. 40 in [14]). Also, the genus *Cretophasmomimoides* is said to differ from other similar genera after its reduced MA (Gorochov [15]'s 2MA_1_). However, similar alterations are a common case in the extant Phasmatodea (OB, pers. obs. and in prep.). Additionally, data on many genera possessing a simple CuA+CuPaα are very limited. Provided these issues, we propose to assign the material to the genus *Cretophasmomima*, but plainly recognized as a rag-bag.

We found no described susumanioid species possessing the wing coloration pattern observed in the new material. Therefore the erection of a new species is well granted.

## Discussion

### *Cretophasmomia melanogramma* is a stem-Phasmatodea

As pointed out in the Introduction, data on the morphology of terminalia of fossil species proved decisive for the identification of susumanioid insects as stem-Phasmatodea: an operculum was described by Ren [12] in three members of the Hagiphasmatidae, a vomer was described by Nel and Delfosse [19] in a Susumaniidae (Figure 1), and an operculum and male forceps-like processes strikingly similar to those of some extant taxa were described by Shang et al. [23] in Susumaniidae.

Material of *Cretophasmomima melanogramma* provides additional evidence for the filiation of susumanioid insects with Phasmatodea. First, females possessed an operculum (Figure 4D–E). Second, all specimens present a Y-shaped dark area in the basal quarter of the forewing. A particular structure occurs in forewings of all extant Phasmatodea in a similar location, and can be referred to as the ‘shoulder pad’ (‘knob-like eversion’ in [17]). It encapsulates the voluminous hind base at rest, and is commonly sclerotized (Figure 5). In some extant species it is converted into a prominent spine [36]. Provided its location, shape and coloration, there is no doubt that the Y-shaped dark area observed in *Cretophasmomima melanogramma* is a ‘shoulder pad’, diagnostic of Phasmatodea. This particular trait could be interpreted as an apomorphic character of a subset within Holophasmatodea ( = total-Phasmatodea; [21], [37]), including at least some stem-Phasmatodea, and extant forms.

A vomer could not be detected in males of *Cretophasmomima melanogramma* (Figure 4F). Similarly, there is no clear evidence of the occurrence of well-developed forceps-like extensions of the 10^th^ abdominal tergum, documented in many members of extant Phasmatodea [21] as well as in a Jurassic susumanioid ([23], and in *Renphasma sinica* (Figure 1). The broad male cerci can also be considered unusual with respect to the morphology of other susumanioid, but is not uncommon among extant phasmid insects [21]. This configuration indicates that, early in their known history, stem-Phasmatodea already displayed a remarkable diversity of their external genitalic morphology.

The twig-like appearance of phasmatodeans is mainly achieved by elongation of specific body parts such as the meso- and metathorax, whereas the prothorax always remains short (unlike in other twig-shaped polyneopterans, i.e. mantodeans and certain caeliferans, e.g. Proscopiidae). In *Cretophasmomima melanogramma* the prothorax is the shortest of the three thoracic segments with equally sized mid and hind legs inserted near the hind margin of the corresponding segment, a typical situation for phasmatodeans [38].

The vast majority of extant forms (and some of their close fossil relatives) pertains to the Euphasmatodea, a clade characterized by a set of derived traits [21], [37]. One character is the basally curved fore femora, which form a notch for the head when the legs are longitudinally aligned during daytime catalepsy (adaptive stillness; [21]). This character has been documented in fossil forms [2]. The fore femora of all specimens of *Cretophasmomima melanogramma* are straight. The most parsimonious interpretation here is the primary absence of this trait, although straight fore femora occur secondarily in some ground-dwelling or aposematically colored diurnal species [3], [21], [39], [40]. However *Cretophasmomima melanogramma* does not show any affinity to members of the corresponding lineages. In other words, we assume that *Cretophasmomima melanogramma* exhibits the plesiomorphic condition in respect to Euphasmatodea and, consequently, cannot be placed within the latter.

This conclusion receives further support from traits retained in the mouthparts: *Cretophasmomima melanogramma* possesses a mandible with several prominent incisivi, which are reduced in the majority of extant euphasmatodeans, with the exception the Chilean *Agathemera* [41]. In addition, the lacinia of *Cretophasmomima melanogramma* bears two apical teeth, representing the plesiomorphic condition among neopteran insects, whereas in the maxillae of Euphasmatodea the lacinia possesses three teeth at its apex [21], [41], [42]. In these respects, viz. the presence of well-developed incisivor lobes and two lacinial teeth, *Cretophasmomima melanogramma* largely resembles the condition found in *Timema* [20].

### Sexual size dimorphism and copulation

Sexual size dimorphism (SSD) is a very widespread phenomenon among insects and is commonly female-biased, viz. females are larger than males [43], [44]. Orthopterans and phasmatodeans are no exception to this rule, as their SSD is almost exclusively female-biased, dramatically in some cases [38], [45], [46]. The higher fecundity exhibited by larger females [47] is commonly advocated as one of the major selective pressures favoring female-biased SSD [48].

As for Phasmatodea, particular aspects of copulation also have implications on the SSD [46]. A male often monopolizes a female by hanging on its back. Pairings, that do not necessarily involve intromission, are notable for their exceptional duration [49]. This practice of post-insemination association, known as mate-guarding [50], reduces the chances of a mated female to accept sperm from another male. It would reinforce a female-biased SSD, as carrying a smaller clinging male is less energy-demanding for a female. On the other hand, on a general basis, larger males are more successful at securing resources, such as females [48]. Although elaborate male clasping organs, documented in many phasmatodean species [21], appear to be an effective mean to prevent attachment by a competitor, males can also engage in fights, in which the larger ones are more likely to succeed [46].

As for *Cretophasmomima melanogramma*, its SSD is only moderately female-biased, with equal wing size in both sexes. Additionally, males apparently did not possess prominent clasping organs that would be effective at maintaining female terminalia when facing the approach of a competing male. These observations and the selective context led us to conjecture that *Cretophasmomima melanogramma* males did not engage in mate-guarding. The current lack of data on SSD of close relatives of *Cretophasmomima melanogramma* makes it difficult to conclude on the polarity of this set of traits, but it likely represents a plesiomorphic condition in regard of extant forms.

Beyond size dimorphism, extant phasmatodeans often exhibit strong sexual dimorphism regarding the extent of wing development, in which macropterous males regularly have large brachypterous, or even fully apterous, females [36], [38], [51]. The overall similarity of both sexes in *Cretophasmomima melanogramma* certainly illustrates another plesiomorphic condition in regard of extant phasmatodeans.

### Leaf mimicry

The Early Cretaceous ecosystem known as ‘Jehol biota’ is exceptionally well documented [52]. The available data on the flora and fauna that co-existed with *Cretophasmomima melanogramma*, and data on the morphology and anatomy of this species, allow inferences on its performance at crypsis.

Virtually all extant stick- and leaf-insects are renowned for their performances at crypsis, mostly visual-based ([53]; among other authors). Body and wing coloration and shape play a central role. However, as for *Cretophasmomima melanogramma*, there is no known counterpart to its peculiar wing coloration. Positioned at rest, the wings, and their straight and parallel dark lines occurring along the whole forewings and hind wing apices, must have produced a tongue-like shape covered with multiple, longitudinal, continuous lines, concealing the abdomen. It should be noticed that the colored area of hind wings (namely, the apices) correspond to the area left uncovered by forewings at rest.

A tongue-shaped outline and the occurrence of multiple longitudinal marked lines is documented in leaves of a number of fossil plants occurring in the same locality as *Cretophasmomima melanogramma*, such as those of the *Baiera*-type and the *Lindleycladus*-type [54]. The corresponding plants have ginkgophytan and conipherophytan affinities, respectively [54], [55]. However, our survey revealed more striking resemblance with *Membranifolia admirabilis* Sun and Zheng in Sun, Zheng, Dilcher, Wang and Mei, 2001 (Figure 6), a comparatively rare remain in the Jehol biota. It must be noted here that the actual affinities of the plant bearing the *Membranifolia admirabilis* organ are not clear, and so is the nature of the organ itself. The general aspect recalls *Baiera* leaves, suggesting relationships with the Gingkophytes. However, because of some characters not commonly seen in *Baiera*, it has also been suggested that *Membranifolia admirabilis* could be a part of a reproduction organ of a plant related to Bennettitales or Gnetales [54]. In any case *Membranifolia admirabilis* was a leaf-shaped plant organ.

**Figure 7:**
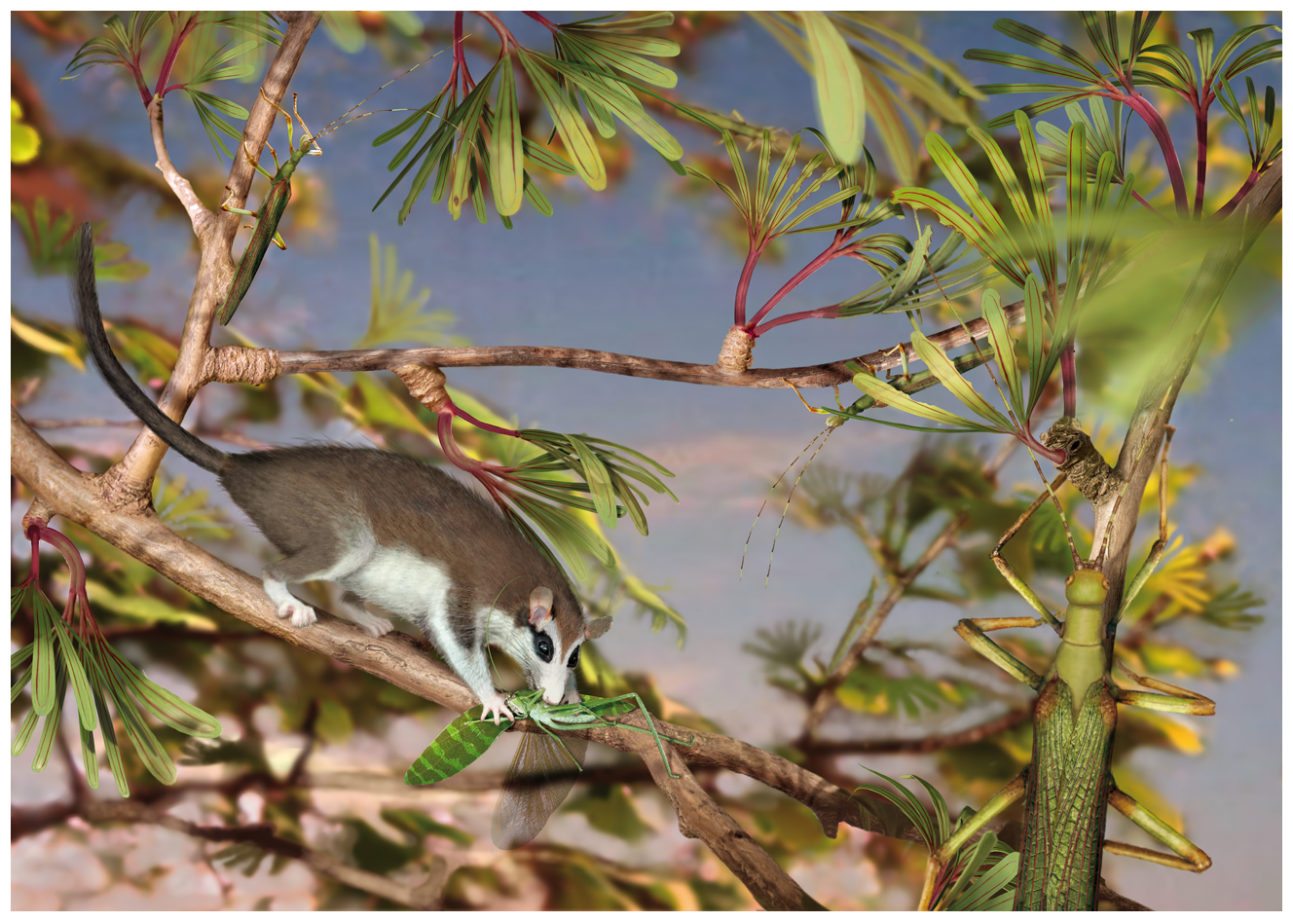
Live reconstruction of *Cretophasmomima melanogramma* Wang, Béthoux and Ren sp. nov. (several exemplars) among *Membranifolia admirabilis* Sun and Zheng, 2001 in Sun, Zheng, Dilcher, Wang and Mei, 2001 (interpreted as Gingkophyte leaf organ). A less camouflaged early orthopteran, *Parahagla sibirica* Sharov, 1968, is captured by the insectivorous *Eomaia scansoria* Ji, Luo, Yuan, Wible, Zhang and Georgi, 2002, one of the earliest eutherian mammals. Reprinted under a CC BY license, with permission from S. Fernandez.

The corresponding leaf-shaped structures are deeply dissected, with three dichotomies (Figure 6). Each of the resulting lobes is long and narrow, and provided with a pair of marked longitudinal veins. The coloration of the rest of the lamina is hardly distinguishable from that of the embedding rock matrix, suggesting that it was made of a thin and somewhat translucent membrane. Similarities with wing of *Cretophasmomima melanogramma* extend to size (compare Figures 2, 3 and Figure 5, provided at the same scale). All these aspects indicate that wing markings observed in *Cretophasmomima melanogramma* would have efficiently concealed the animal within a cluster of *Membranifolia admirabilis* organs (Figure 7 –under a ‘Gingkophyte-leaf hypothesis’).

The ‘model plant’ of extant mimicking Phasmatodea is commonly the plant also used as food resource. Assuming that *Membranifolia admirabilis* belongs to a Ginkgophyte, it must be noticed that there is no extant Phasmatodea known concealing or foraging on *Ginkgo biloba* Linnaeus, 1771, the only extant representative of the group. The impressive chemical defenses of the plant, and prompt response to herbivore attacks [56], might explain this situation. However, Ginkgophytes were flourishing during the Jurassic and the Early Cretaceous [55], and leaf marginal feeding is documented in the group, as early as in the Triassic [57]. Assuming that *Membranifolia admirabilis* belonged to a Bennettitale or a Gnetale, the fact that both groups have been proposed as sister-groups of angiosperms [58], and that the latter group is the main food resource of extant Phasmatodea, suggest that any of these could have been equally palatable to *Cretophasmomima melanogramma*. Last but not least, studies on *Timema*, the extant sister group of all remaining crown-phasmatodeans, reveal several shifts in host plant, within a few million years, and across plant lineages that are phylogenetically distant [59]. In summary, neither the extant situation nor uncertainty on the actual affinities of the plant bearing *Membranifolia admirabilis* organs preclude on the possibility that *Cretophasmomima melanogramma* used the corresponding plant as a food resource (in addition to imitating the appearance of its leaves, or leaf-shaped organs).

A number of potential insect predators were reported from the Jehol biota. Several enantiornithine birds are depicted as small-sized, arboreal, and insectivorous [60]. The diverse mammal fauna of the Jehol biota also includes potential predators, such as *Eomaia scansoria* Ji, Luo, Yuan, Wible, Zhang and Jianping, 2001 (represented on Figure 7), and *Sinodelphys szalayi* Luo, Ji, Wible and Yuan, 2003, both viewed as small, agile branch-walking insectivores [61]. The corresponding diversifications probably triggered the acquisition of primary defense mechanisms such as leaf mimicry, observed in *Cretophasmomima melanogramma*.

## Conclusion

Based on the combination of observed characters, viz. presence of ‘shoulder pads’ in the forewings, aspect ratios of thoracic segments and legs, unsegmented cerci, we infer that *Cretophasmomima melanogramma* is a genuine stem-Phasmatodea. The fossil stick insect already possessed leaf (or leaf-shaped organ) mimicking capabilities, but retained a number of putative plesiomorphic traits in regard to extant forms, such as presence of mandibular incisivi, two lacinial teeth, straight fore femora, well-developed long forewings in both sexes and a low degree of sexual size dimorphism that is only moderately female-biased. This new record suggests that leaf mimicry predated the appearance of twig and bark mimicry in phasmatodeans. Additionally, it complements our growing knowledge of the early attempts of insects to mimic plant parts [62]–[64].
